# Proposed correlation of structure network inherited from producing techniques and deformation behavior for Ni-Ti-Mo metallic glasses *via* atomistic simulations

**DOI:** 10.1038/srep29722

**Published:** 2016-07-15

**Authors:** M. H. Yang, J. H. Li, B. X. Liu

**Affiliations:** 1 Key Laboratory of Advanced Materials (MOE), School of Materials science and Engineering, Tsinghua University, Beijing 100084, China

## Abstract

Based on the newly constructed *n*-body potential of Ni-Ti-Mo system, Molecular Dynamics and Monte Carlo simulations predict an energetically favored glass formation region and an optimal composition sub-region with the highest glass-forming ability. In order to compare the producing techniques between liquid melt quenching (LMQ) and solid-state amorphization (SSA), inherent hierarchical structure and its effect on mechanical property were clarified *via* atomistic simulations. It is revealed that both producing techniques exhibit no pronounced differences in the local atomic structure and mechanical behavior, while the LMQ method makes a relatively more ordered structure and a higher intrinsic strength. Meanwhile, it is found that the dominant short-order clusters of Ni-Ti-Mo metallic glasses obtained by LMQ and SSA are similar. By analyzing the structural evolution upon uniaxial tensile deformation, it is concluded that the gradual collapse of the spatial structure network is intimately correlated to the mechanical response of metallic glasses and acts as a structural signature of the initiation and propagation of shear bands.

Bulk metallic glasses (BMGs) have attracted considerable interests due to their fundamental scientific significance as well as great potential for engineering applications^1^,^2^,^3^,^4^,^5^. To date, a great number of multicomponent BMGs have been obtained through a variety of powerful producing techniques, such as liquid melt quenching (LMQ)^6^, solid-state amorphization (SSA)^7^ and ion beam mixing (IBM)^8^. The SSA in the present study refers to solid solution models, *i*.*e*., the underlying physics of metallic glass formation is the spontaneous collapse of the crystalline lattice while the solute concentration exceeds a critical value. In order to facilitate the production of BMGs, the first priority issue is to clarify the formation mechanism, which could serve as guidance in synthesizing the desired glassy alloys^9^. In this respect, a terminology named glass-forming ability (GFA) has long been used in describing the easiness or difficulty of metallic glass formation. In practice, the GFA is usually evaluated by the maximum size (*D*_*max*_) of the obtainable BMGs, or the minimum cooling speed (*R*_*c*_) to produce BMGs by LMQ, *e*.*g*., copper model casting^10^. It follows that the smaller *R*_*c*_ or the larger *D*_*max*_ is, the better GFA of the alloy should be. Meanwhile, a negative correlation of GFA with the critical dosage, *i*.*e*., minimum ion-dosage, is recently proposed for amorphous alloys formed by IBM, *i*.*e*., the lower critical ion-dosage (*D*_*c*_), the better GFA, suggesting that *D*_*c*_ could serve as an indicator to the GFA of these alloys. The proposal helps to bridge the gap of experimental data between different producing methods, *e*.*g*., making it possible to apply the IBM results to designing alloy compositions when producing BMGs by LMQ^11^. However, there is still lack of relevant research on the atomic-level structure and structure-property relationship of BMGs to compare the producing methods between LMQ and SSA. The present work attempts to solve this problem based on atomistic modeling of Ni-Ti-Mo metallic glasses produced by both the liquid melt quenching and solid solution models.

Previously, several empirical rules or criteria have been proposed and served as guidelines for the design of favored compositions for glass formation, of which the frequently cited are deep eutectic rule, size difference rule, Miedema’s model and so on^12^,^13^,^14^. However, these predictions are unsatisfactory when comparing with the experimental results, and their starting bases have some restrictions in well reflecting the internal characteristics of the alloy system concerned. As a result, the key is to seek for a valid starting base and further develop an approach capable of clarifying the metallic glass formation in the specific alloy system. From a physical viewpoint, the interatomic potential of an alloy system is able to reasonably describe the major interactions involved in the system. Therefore, if a realistic interatomic potential is constructed, most of the physical and chemical properties of the system, including those related to the BMGs, can be deduced through relevant computations and simulations^15^.

The atomic-level structure and structure-property relationship of BMGs has long been a significant issue in the fields of materials science and solid-state physics^16^,^17^. It has been revealed that the distinct atomic sizes and multiple chemical interactions among various types of atoms in BMGs do result in the formation of short-range order (SRO), regarding as the building blocks of the three-dimensional glassy structure^18^,^19^. However, how these local clusters interconnect to achieve efficient packing remains a mystery, and much effort has been made to unravel the higher hierarchy of order, *i*.*e*., medium-range order (MRO) or a spatial network that fulfills the real space^20^,^21^. For instance, it was revealed that the interpenetrating linkages of icosahedra exhibit strong correlation in space and tend to aggregate and form string-like networks^22^,^23^,^24^. Although the emphasis in present studies has been laid on the interpenetrating linkages, *i*.*e*., the volume sharing linkages among the clusters, it is also important to elucidate the roles of other noninterpenetrating linkages, *i*.*e*., the vertex, edge, and face sharing linkages, to reveal how these various structural characteristics are collectively hybridized in constituting the densely packed network in BMGs^25^,^26^,^27^,^28^. Therefore, the characteristics of the spatial network is important to provide an explicit picture of the hierarchical structure in BMGs and further clarify the structure-property relationships.

Nowadays, many efforts have been devoted to studying the effects of the element addition on the structure-property relationships of the alloy system. Chun *et al*.^29^ has reported that with the addition of 4.8% carbon to Ni-Ti metallic glasses, the uniaxial elastic constants and Young’s modulus are increased by 6.5% and 12.0%, respectively. In the present work, we also aim to study the effects of Mo addition on the metallic glass formation, atomic structure and mechanical properties of the Ni-Ti binary system. The paper proceeds as follows: (I) a new long-range *n*-body potential was first constructed for the Ni-Ti-Mo ternary system, and the relevant results are presented in the supplementary material I; (II) based on the solid solution method, a series of Molecular Dynamics (MD) and Monte Carlo (MC) simulations were conducted to investigate the glass formation region (GFR) and the optimal compositions for the Ni-Ti-Mo system in supplementary material II; (III) the characteristics of hierarchical atomic structure obtained from LMQ and SSA were analyzed in terms of their connectivity and rigidity; (IV) the mechanical responses and associated structural evolutions under the uniaxial tensile deformation are monitored and compared between both producing techniques.

## Results and Discussion

### Hierarchical atomic structure of Ni-Ti-Mo metallic glasses

#### Local atomic structure

**Figure 1:**
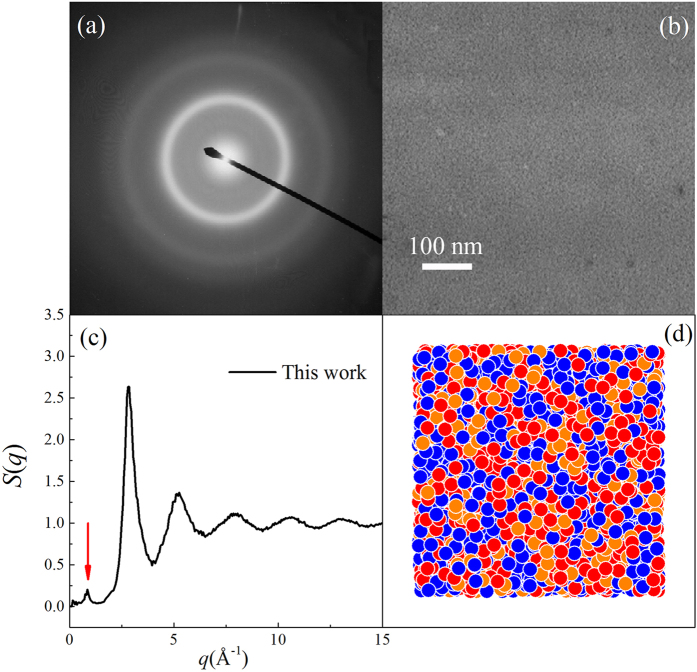
The SAD pattern (**a**) and the corresponding bright field image (**b**) of the (NiTi)_80_Mo_20_ alloy obtained by IBM after irradiation to the dose of 7 × 10^15^ Xe^+^/cm^2^. The prepeak is marked by red arrow in the structure factor. Structural factors (**c**) and atomic position projections (**d**) for the amorphous state (NiTi)_80_Mo_20_.

We now present the simulation results of the Ni-Ti-Mo system. After adequate MD time steps at a given temperature, the structures of simulation models generally exhibit three states: a crystalline state, an ordered-disordered transitional state, and an amorphous state. We take the (NiTi)_80_Mo_20_ alloy as an example. Figure 1(a,b) show the selected area diffraction (SAD) patterns and corresponding bright field image of (NiTi)_80_Mo_20_ alloy^11^. One can see the diffused halos without diffraction rings obtained in Fig. 1(a) show a typical amorphous configuration without solid solutions, confirmed by the uniform gray matrix exhibited in Fig. 1(b). Meanwhile, the structure factor *S*(*q*) and the projections of the atomic positions for (NiTi)_80_Mo_20_ alloy are presented in Fig. 1(c,d). It can be seen that the original crystalline lattice has spontaneously collapsed and turned into a completely amorphous state, exhibiting a short-range order and long-range disorder feature. As shown in Fig. 1(c), the prepeak in the reciprocal space should correspond to certain medium-range correlations in real space^16^. The first peak of the *S*(*q*) appears at the *q*_1_ = 2.80 Å^−1^, while the second peak and third peak appear at the *q*_2_ = 5.25 Å^−1^ and *q*_3_ = 7.85 Å^−1^, respectively. For comparison, one can see from Fig. 1(a) that three haloes correspondingly emerged in the SAD patterns. Based on the camera length and wave length, the average diameters of the three haloes can be transferred to the wave vectors *q*, *i*.*e*., *q*_*a*_ = 2.8188 Å^−1^, *q*_*b*_ = 5.2488 Å^−1^, and *q*_*c*_ = 7.6788 Å^−1^, respectively. The deviations of *q*_*a*_ compared with *q*_1_, *q*_*b*_ compared with *q*_2_, and *q*_*c*_ compared with *q*_3_ are all lower than 3%, indicating that the structure prediction of the Ni-Ti-Mo system by MD simulations are consistent with the experimental results.

The local atomic structures in the MGs were analyzed in terms of the types and degrees of short-range orders. In order to characterize the topological SROs, two methods were jointly applied to extract structural information from different perspectives, *i*.*e*., Honeycutt-Anderson pair analysis and Voronoi tessellation methods. Firstly, we present the results of Honeycutt-Andersen pair analysis. To perform a detailed investigation, the local structures of (NiTi)_80_Mo_20_ MGs obtained from LMQ and SSA are presented in Table 1. It lists the 1551 index (regarded as the icosahedral ordering), the 1541 and 1431 indices (*i*.*e*., distorted icosahedral ordering), the 1441 and 1661 indices (*i*.*e*., the *bcc* ordering), and the 1421 and 1422 indices (*i*.*e*., the *fcc* ordering and *hcp* ordering). With the Mo concentration increases, it can be seen that the fraction of bond pairs with indices of 1551, 1541, 1661 and 1441 increases for both producing techniques, whereas the fraction of bond pairs with indices of 1431 remains stable. By comparison, a large number of the 1551 bond pairs are found in the local configurations of (NiTi)_80_Mo_20_ MGs, indicating that the fivefold bonds and triangulated faces are indeed encouraged in the metallic glasses. Meanwhile, the distorted fivefold bond pairs with indices of 1541 and 1431 are dominant in these alloys, covering a fraction of ~35%. In addition, the *fcc*- or *hcp*-like bond pairs with indices of 1421 and 1422 cover a relatively lager fraction in alloys. Therefore, the local atomic structure of these systems embodies imprints of both the icosahedral, *fcc*- or *hcp*-like configurations. By independently analyzing the atomic configurations derived by LMQ and SSA, it is revealed that they exhibited no pronounced differences in the local atomic structure.

**Figure 2:**
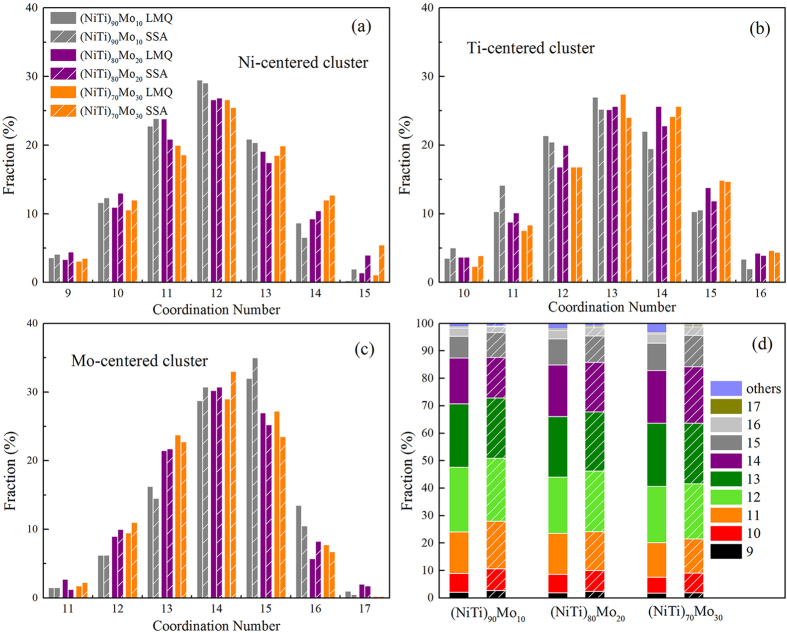
Distributions of the coordination number (CN): (**a**) Ni-centered, (**b**) Ti-centered, (**c**) Mo-centered, and (**d**) total of (NiTi)_90_Mo_10_, (NiTi)_80_Mo_20_, and (NiTi)_70_Mo_30_ MGs obtained by LMQ and SSA.

In addition to Honeycutt-Anderson pair analysis, Voronoi tessellation analysis is a more three-dimensional (3D) approach since it provides a more complete geometrical construction of a central atom to its neighboring atoms. To investigate the effects of the producing methods between LMQ and SSA on the local atomic structure, the evolution of the CN distributions surrounding Ni-centered, Ti-centered, Mo-centered of (NiTi)_90_Mo_10_, (NiTi)_80_Mo_20_, and (NiTi)_70_Mo_30_ MGs were calculated and analyzed in Fig. 2(a–c). It is seen that Ni-centered clusters are centered in CN = 12, Ti-centered clusters are centered in CN = 13 and 14, whereas a relatively concentrated range is observed for the CNs of Mo-centered clusters, mainly varying from 14 to 15. Considering the larger concentration of Ti or Ni atoms, they are supposed to serve as solvents and neighbors for Mo atoms. Therefore, it is plausible to expect that Mo atoms could more readily adopt a favored local packing at their preference, whereas Ti or Ni atoms would experience more flexible local environments. Meanwhile, we have correspondingly analyzed the atomic configurations obtained by LMQ and SSA, and found that they exhibit no intrinsic differences in both of the producing methods. However, there is a trend that the LMQ method produces more low-CN or less high-CN clusters than SSA. Such changes are further reflected by the overall CN distributions for (NiTi)_90_Mo_10_, (NiTi)_80_Mo_20_, and (NiTi)_70_Mo_30_ MGs in Fig. 2(d).

**Figure 3:**
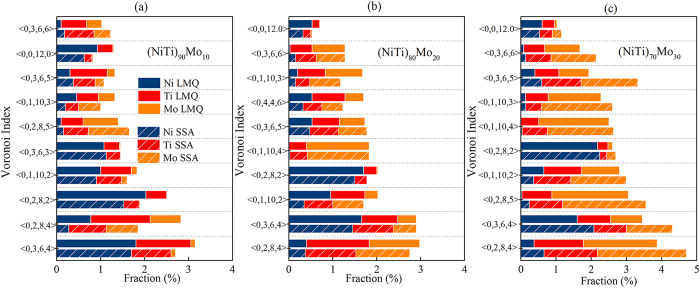
Populations of the dominant coordination clusters of the obtained MGs: (NiTi)_90_Mo_10_ (**a**), (NiTi)_80_Mo_20_ (**b**) and (NiTi)_70_Mo_30_ (**c**).

The fractions of the dominant Voronoi polyhedra around the constituent elements of (NiTi)_90_Mo_10_, (NiTi)_80_Mo_20_, and (NiTi)_70_Mo_30_ MGs obtained by LMQ and SSA were then calculated and illustrated in Fig. 3(a–c), respectively. The dominant polyhedra in (NiTi)_90_Mo_10_, (NiTi)_80_Mo_20_, and (NiTi)_70_Mo_30_ MGs, aligned from left to right, are indexed as 〈0, 2, 8, 4〉, 〈0, 3, 6, 4〉, 〈0, 2, 8, 2〉, 〈0, 1, 10, 2〉, regarded as distorted icosahedra. It is interesting to note that the icosahedron 〈0, 0, 12, 0〉 is not the most abundant index, but it is within the top 10 and have lowest energies than other distorted icosahedra^30^. It is known that icosahedron or distorted icosahedra play an important role in stabilizing the structure of the metallic glass-forming system because of the efficient atomic packing and energy minimization^31^,^32^. This is consistent with the results presented in Table 1 that 1551, 1431, and 1541 indicating icosahedron or distorted icosahedra has a rather high total fraction in the (NiTi)_100−*x*_Mo_*x*_ MGs.

#### Spatial connectivity between the icosahedron or distorted icosahedra

**Figure 4:**
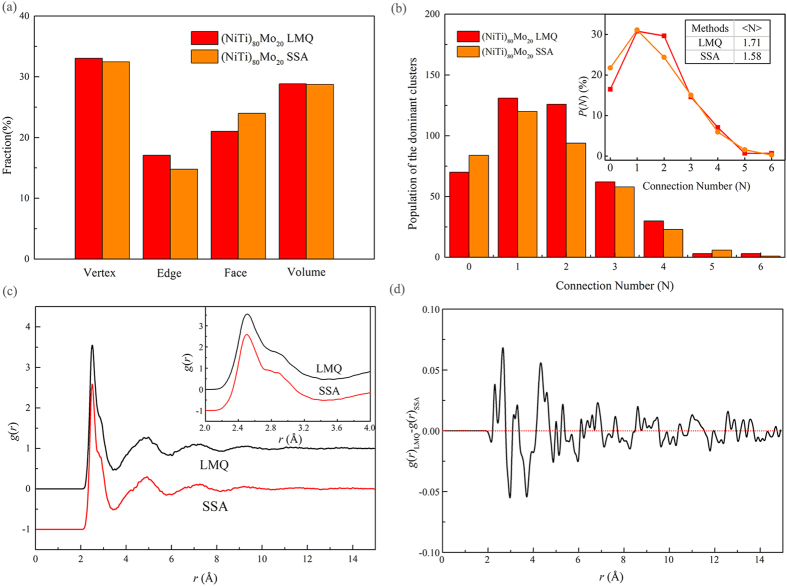
Populations of the four different linkages with the neighboring dominant clusters (**a**). Variations in the fractions of the dominant clusters and different connection numbers in (NiTi)_80_Mo_20_ MGs, and the liquid melt quenching MGs have a higher degree of medium-range ordering, due to its higher population and 〈*N*〉 (**b**). The pair-correlation function *g*(*r*) curves of (NiTi)_80_Mo_20_ MGs obtained from LMQ and SSA in a range of atomic distance (**c**) and their difference curve (**d**), respectively. Enlarged *g*(*r*) curves of both MGs in a range of atomic distance 2–4 Å are also presented.

To present a complete picture of the hierarchical structure in the Ni-Ti-Mo MGs, the spatial structure network formed among the icosahedron or distorted icosahedra of (NiTi)_80_Mo_20_ MGs obtained by LMQ and SSA were analyzed and the relevant schematic diagrams are presented in the supplementary material IV. According to the Voronoi analysis, the majority (over 95%) of the dominant clusters of (NiTi)_80_Mo_20_ MGs obtained by both producing methods exist in the form of linked clusters, rather than isolated ones. By analyzing the configuration of the spatial structure network in (NiTi)_80_Mo_20_ MGs, the respective fractions of the vertex, edge, face and volume linkages were calculated and exhibited in Fig. 4(a). It can be seen that the four different linking patterns in (NiTi)_80_Mo_20_ MGs obtained by LMQ and SSA are almost the same, indicating that no pronounced differences of the cluster connectivity are found for both producing methods. Of the four different cluster linking patterns, the volume-sharing pattern, also termed as the interpenetrating connection, is of special interests. It has been reported that the volume linkage has been revealed to be the lowest average potential energy and smallest average atomic volume^33^,^34^. Therefore, the volume linkage is likely to be the most important type of the four different linkages to form the spatial structure network. Comparing to the interpenetrating connection, the noninterpenetrating linkages, *i*.*e*., vertex, edge and face linkages, consolidating the structural and energetic stability of the network^35^. As a result, the various cluster interactions contribute collectively to achieve the stabilized and efficient filling of space.

After deciding on the important cluster sharing scheme (the interpenetrating type), the next characteristic to examine is how well and extensive the volume-sharing linkage, *i*.*e*., how many cluster neighbors the dominant cluster is connecting with. This number is defined as the connection number, *N*, and the spread degree of centered atoms is characterized by a distribution function *P*(*N*). To show the connectivety of the dominant cluster *via* volume sharing, the average value 〈*N*〉 calculated by 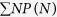 would be a good indicator:


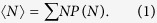


From Fig. 4(b), one can see that the population of the icosahedron or distorted icosahedra with various *N* in (NiTi)_80_Mo_20_ MGs obtained by LMQ and SSA. Although the connection number of the icosahedron or distorted icosahedra shows no pronounced differences between two producing techniques, there also exists some difference. Compared to (NiTi)_80_Mo_20_ MGs obtained by LMQ, the population of isolated clusters (*N* = 0) obtained by SSA is higher, whereas LMQ not only produces more clusters participating in the formation of the spatial structure network, but also has a higher cross-linking degree as evaluated in terms of 〈*N*〉. This means that the liquid melt quenching method will make a relatively more ordered structure with a higher atomic packing density and lower energy state.

To give more detailed information for atomic packing, the pair-correlation function *g*(*r*) is commonly used to identify difference of atomic-level packing between LMQ and SSA. To calculate the pair-correlation function *g*(*r*), each atom was imagined to be at the center of a series of concentric spheres. The total pair correlation function, *g*(*r*), could be obtained by the following formula:


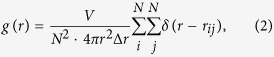


where *V* is the volume of the calculated cell, *N* is the total number of atoms, and *N*_A_ and *N*_B_ are the number of A and B atoms. Δ*r* is the distance interval in the calculation. The function *δ*(*t*) equals one when −0.5Δ*r* < *t* < 0.5Δ*r* and equals zero otherwise. Figure 4(c,d) present the *g*(*r*) curve of (NiTi)_80_Mo_20_ MGs obtained by LMQ and SSA in a range of atomic distance and their difference curve, respectively. As shown in Fig. 4(c), the *g*(*r*) curves of both MGs are similar with a few small humps in the distance, and the enlarged inset in a range of atomic distance 2–4 Å illustrates that the first peak split into two peaks. In addition, the fine difference between the *g*(*r*) curves obtained from LMQ and SSA are presented in Fig. 4(d). Compared to the MGs obtained by SSA, the liquid melt quenching MGs show a higher *g*(*r*) in the first two coordination shells of *r* ~ 2.3–4.8 Å. The relative change in the highest peak height is about 15%. In higher coordination shells of *r* ~ 4.8–8.3 Å, *g*(*r*) in solid-state amorphization MGs becomes higher. As atomic distance *r* increases, no obvious difference was detected. Therefore, the MGs obtained by LMQ has higher coordination numbers in the range of *r* ~ 2.3–4.8 Å and lower ones in the range of *r* ~ 4.8–8.3 Å than those obtained by SSA. Under liquid melt quenching treatment, the atomic configuration in MGs are packed even denser in the first two coordination shells and less in the third coordination shells as compared to those for solid-state amorphization MGs^36^,^37^.

### Structural signature of shear localization

#### Stress-strain curves

It is well known that BMGs lack the tensile ductility, considered as one of the main problem during the wide practical applications^38^. Therefore, an in-depth understanding of the elastic-plastic behavior for MGs is needed. In addition, the plastic behavior in metallic glasses distinguishes from their crystalline counterparts and thus the relationship between atomic-microstructure and properties remain one of the top research interests during the recent decades^39^. Because of the wide span of time and length-scales in the mechanical behaviour, it is rather difficult to establish the relationship between the macroscopic property and the microscopic structure of MGs^40^,^41^. As a result, large scale MD simulations were performed to study the mechanical behaviour of the Ni-Ti-Mo MGs and corralate it to the internal structures.

**Figure 5:**
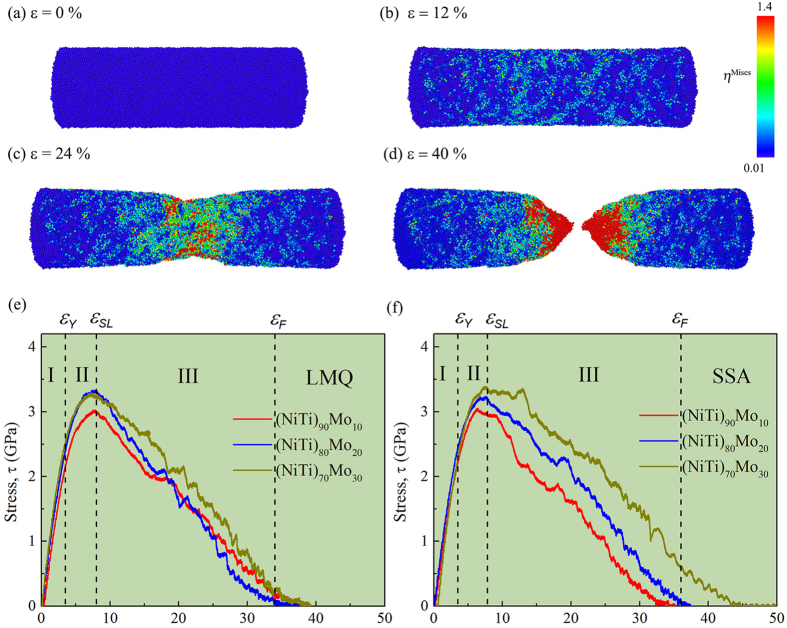
Schematic diagram at different strains (**a**–**d**) of the (NiTi)_80_Mo_20_ MGs obtained by LMQ containing ~155000 atoms at 100 K. Simulated stress-strain curves under uniaxial tensile loading for (NiTi)_90_Mo_10_, (NiTi)_80_Mo_20_, and (NiTi)_70_Mo_30_ MGs containing ~155000 atoms at 100 K obtained by LMQ (**e**) and SSA (**f**).

To further illustrate the deformation behavior, we display the schematic diagram at four different strain percentages in Fig. 5(a–d), and the atoms are colored based on the local atomic shear strain 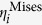. As shown in Fig. 5(a), we present the initial condition, where all the atoms are around their initial positions and this behavior holds until about 4%. After that, some atoms begin to cross the threshold of 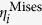 shear strain and become shear deformation zones (STZs). When the strain increases to 12% in Fig. 5(b), the clusters of atoms with the local atomic shear strain greater than the threshold appear on the surface. As seen in Fig. 5(c), these STZs propagate and nucleate, becoming the shear band (SB) in the middle part of the sample. Eventually, the SB develops and fails in Fig. 5(d).

As shown in Fig. 5(e,f), we present the stress-strain relations of (NiTi)_90_Mo_10_, (NiTi)_80_Mo_20_, and (NiTi)_70_Mo_30_ MGs obtained by LMQ and SSA. The deformation behaviors can be divided into three distinct regimes: (i) for the initial elastic deformation stage at strains [0, *ε*_*Y*_), stress increases linearly with strain before reaching an elastic behavior up to 4%; (ii) for the homogeneous deformation zone at [*ε*_*Y*_, *ε*_*SL*_), the curves deviate from the initial linearity until the maximum stress (denoted as overshoot stress (*σ*_*over*_) is reached, and samples in this regime experience considerable uniform elongation; (iii) for the inhomogeneous zone at [*ε*_*SL*_, *ε*_*F*_), the deformation is found to concentrate in the necking zone because of the formation and propagation of the SB, and the samples experience non-uniform elongation around the initial necking area until it finally separates into two parts. With increasing Mo concentration in (NiTi)_100−*x*_Mo_*x*_ MGs, one can see from Fig. 5(e,f) that both *σ*_*over*_ and the failure strain *ε*_*F*_ obtained from SSA increase markedly, whereas the LMQ method makes both *σ*_*over*_ and *ε*_*F*_ lack sensitivity to the Mo concentration. Specifically, the overshoot stress *σ*_*over*_ reflects the intrinsic resistance to flow initiation of the original MG samples, or the stress required to achieve rejuvenation^42^ at the given loading conditions (strain rate, temperature, and sample morphology, etc.).

**Figure 6:**
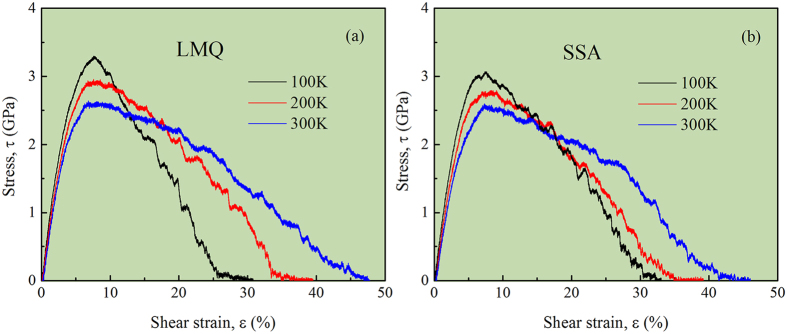
Temperature dependence of simulated stress-strain curves for (NiTi)_80_Mo_20_ MGs containing ~100000 atoms obtained by LMQ (**a**) and SSA (**b**). It should be noted that the simulated stress-strain curves are dependent on the model size or diameter size (see supplementary material III).

It is worth noting that the values of *σ*_*over*_ and *ε*_*F*_, although directly reflecting the particular sample’s intrinsic properties, are dependent on the details of the loading tests. To be more specific, we discuss two important external parameters: temperature and diameter size (see supplementary material III). When the temperature increased from 100 K to room temperature (at 300 K) in Fig. 6, it can be seen that the value of *σ*_*over*_ obtained from both LMQ and SSR methods decreased, while the value of *ε*_*F*_ increased. According to the core equation of cooperative shear model (CSM), written in terms of the shear stress and shear modulus, is given as


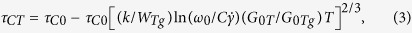


where *τ*_*CT*_ is the yield stress at temperature *T* corresponding to the critical activation rate of STZ relative to the shear rate 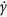, *τ*_*C*0_ is the yield stress without thermal activation (*i*.*e*., a thermal limit at *T* = 0), *ω*_0_ and *C* are constants, and *G*_0*T*_/*G*_0*Tg*_ is the ratio of zero-stress shear moduli at *T* and *T*_*g*_, respectively. This ratio is close to unity because the glass remains in essentially the same configuration after glass transition, and there is only a weak *T* dependence due to thermal expansion. The detailed model derivation can be found in ref. 43. The main conclusion from Eq. (3) is that the yield stress is a function of temperature: lower temperature results in higher yield stress, matching well with the simulation results.

#### Atomic structure based on strain evolution

**Figure 7:**
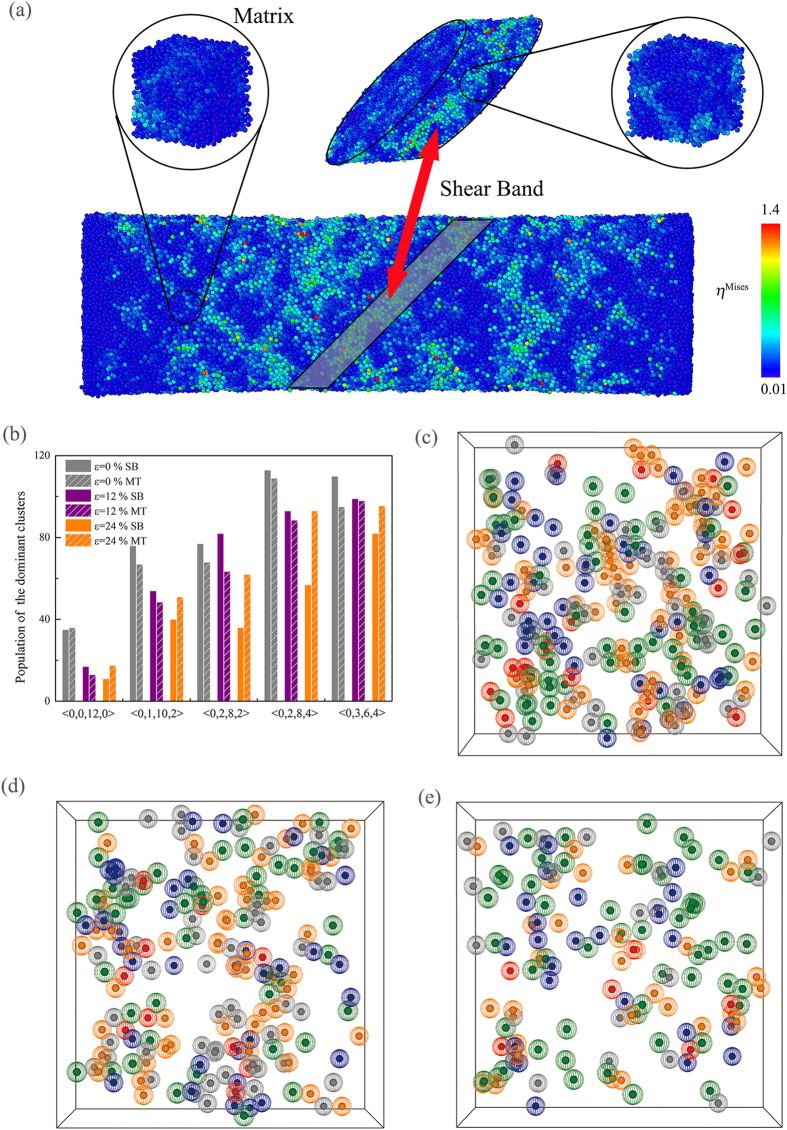
Schematic diagram of how the shear band and the matrix samples were prepared (**a**). The population of the dominant clusters for the shear band and the matrix samples of (NiTi)_80_Mo_20_ MGs obtained by LMQ (**b**), and distribution of the spatial structure network of the dominant clusters: an original state (**c**), 12% shear strain (**d**) and 24% shear strain (**e**). For clarity, only the top half of the simulation box is shown in (**c**–**e**). Red dot surface are for 〈0, 0, 12, 0〉, purple dot surface for 〈0, 1, 10, 2〉, gray dot surface for 〈0, 2, 8, 2〉, orange dot surface for 〈0, 2, 8, 4〉, and green dot surface for 〈0, 3, 6, 4〉.

During the past years, numerous studies have been carried out in order to understand the SB nucleation, and relate them to the defects given in crystalline metals^44^,^45^,^46^,^47^. Especially, it would be desirable to have studies directly correlating the evolution of the SB and matrix structure to the applied strain, allowing us to easily connect the atomic structure evolution to the stress-strain curve. In the present work, we perform a comparative analysis of the atomic structure of the both regions. One can see from Fig. 7(a) a schematic diagram of how the SB and the matrix regions were obtained. At first, we identify the SB by visual inspection, corresponding to the regions inside the black line zone of Fig. 7(a). Afterwards, we extract a slice with high local atomic shear strain, represented by the shaded zone. From this slice, we extract a cube of 20 Å edge containing around 4000 atoms, labeled as “Shear Band” (SB). The same procedure is applied to the matrix system, resulting in a sample with the same volume, labeled as “Matrix” (MT)^48^.

In order to compare the difference between both regions, (NiTi)_80_Mo_20_ MGs obtained by LMQ were analyzed by means of Voronoi tessellation methods, where the atoms on the surface aren’t considered. As shown in Fig. 7(b), it lists the changes of the icosahedron 〈0, 0, 12, 0〉 and distorted icosahedra 〈0, 2, 8, 4〉, 〈0, 3, 6, 4〉, 〈0, 2, 8, 2〉, 〈0, 1, 10, 2〉 of both “Shear Band” and “Matrix” regions according to the applied strain at 0%, 12% and 24%, respectively. At the strain of 0% and 12%, both “Shear Band” and “Matrix” regions present similar percentages of the dominant clusters. When the strain increases to 24%, the “Matrix” region present more percentage of the dominant clusters than the “Shear Band” region, indicating the changes occurred in the SB are more subtle^49^. To directly characterize and visualize the spatial structure network in the “Shear Band”, we present the distribution of the dominant clusters at *ε* = 0%, 12%, and 24%. It can be seen that the higher the shear strain, the lower the concentration of the dominant clusters, and vice versa. When *ε* = 12%, close to the onset of the stress drop in Fig. 5(e), the structure network starts to collapse, allowing the local inelastic deformation sites to percolate and form shear bands. As a result, it is revealed that the deconstruction of the connectivity between short-range orders (SROs) in space are consistent with the formation of shear bands, and the yielding of MG is the process of overcoming the interpenetrated rigidity developed in internal structure^50^. When the spatial structure network continues to fall apart, the network has been destroyed into several discrete patches at *ε* = 24% in Fig. 7(d). Therefore, it can be concluded that the distribution of the spatial structure network changes at the varying deformation stages, and correlates with the mechanical response in the MGs, particularly the initiation and propagation of shear bands.

## Conclusion

A long-range empirical potential is firstly constructed for the Ni-Ti-Mo ternary system, particularly consisting of three constituent metals with different crystalline structures. Based on the newly constructed long-range Ni-Ti-Mo *n*-body potential, the Molecular Dynamics (MD) and Monte Carlo (MC) simulations predict a hexagonal composition region within which metallic glass formation (GFR) is energetically favored. Moreover, the atomistic approach further predicts an amorphization driving force (ADF) for each alloy located within the GFR, and pinpoints the optimal composition sub-region with the highest glass-forming ability (GFA) in the glass formation region.

To compare the producing techniques between liquid melt quenching (LMQ) and solid-state amorphization (SSA), atomic-level structure and its effect on the deformation behavior were investigated for Ni-Ti-Mo MGs by atomistic simulations. It is found that the dominant clusters of (NiTi)_80_Mo_20_ MGs obtained by LMQ and SSA are the icosahedron 〈0, 0, 12, 0〉 and distorted icosahedra 〈0, 2, 8, 4〉, 〈0, 3, 6, 4〉, 〈0, 2, 8, 2〉, 〈0, 1, 10, 2〉. In forming the spatial structure network, the dominant clusters achieve their mutual percolation in space, and the various cluster interactions, *i*.*e*., volume, vertex, edge and face linkages, contribute collectively to achieve the stabilized and efficient filling of space. Furthermore, by monitoring the structural rearrangements during uniaxial tension deformation, it was revealed that the coverage and distribution of spatial structure network evolves at the varying deformation stages, and is closely correlated to the initiation and propagation of shear bands in MGs. The breakdown of the short-range orders (SROs) and their connectivity in space are consistent with the formation of shear bands, promoting the structural understanding of the yielding deformation of MGs. By independently analyzing the atomic configurations and deformation behavior between LMQ and SSA, it is found that they exhibit no pronounced differences in the local atomic structure and mechanical behavior, whereas the liquid melt quenching method makes a relatively more ordered structure and a higher intrinsic strength.

## Methods

### Solid solution model

As the process of producing metallic glasses is always a far-away equilibrium process, the complicated phase cannot nucleate and grow due to the extremely restricted kinetic condition. It follows that the phase competing against the amorphous phase is the solid solution with one of the three simple structures, *i*.*e*., *fcc*, *hcp*, or *bcc*. A great deal of research results from both experimental and theoretical aspects^27^,^51^ have confirmed the above point of view. Therefore, the issue related to glass-formation region of Ni-Ti-Mo system can be transformed to compare the relative stability of the solid solution to its disorder counterpart, *i*.*e*., amorphous alloy.

Based on the constructed Ni-Ti-Mo potential, MD simulations using Large-scale Atomic/Molecular Massively Parallel Simulator (LAMMPS) packages were implemented to study the relative stabilities of Ni-Ti-Mo solid solutions against their amorphous counterparts within the entire composition region of the system. The *fcc* and *hcp* solid solution models contain 4000 (10 × 10 × 10 × 4) atoms, while the *bcc* model consists of 2000 (10 × 10 × 10 × 2) atoms. Periodic boundary conditions were adopted in the three directions. MD simulations in the framework of an isothermal-isobaric ensemble were performed in a time step of 5 femtoseconds. The simulation proceeded at 300 K and 0 Pa for 1 million time steps to achieve a stable state.

Meanwhile, a series of MC simulations were performed to calculate the formation energy of the solid solutions. The corresponding models were built up in the same way as in MD simulations. During the simulations, there exist two types of “moves”: atom displacement and box deformation. Concerning the details of MC simulations, the readers can refer to the recent published paper^51^.

### Liquid melt quenching

In the present study, the (NiTi)_80_Mo_20_ MGs were selected as a principal model alloy. In the following section, (NiTi)_80_Mo_20_ was found to be within an optimal composition sub-region of the Ni-Ti-Mo system, where the amorphization driving force, defined as the energy difference between the amorphous state against that of the competing solid solution counterpart, are larger than those outside the sub-region. In order to compare the structure and its effect on the properties, two other MGs, *i*.*e*., (NiTi)_90_Mo_10_ and (NiTi)_70_Mo_30_, also within an optimal composition sub-region of the Ni-Ti-Mo system, were employed as reference alloys.

According to the constructed Ni-Ti-Mo potential, MD simulations were conducted to three MGs, *i*.*e*., (NiTi)_90_Mo_10_, (NiTi)_80_Mo_20_ and (NiTi)_70_Mo_30_, consisting of 4000 atoms in a cubic box. Periodic boundary conditions were enforced in all three dimensions. The pressure was kept at zero using the Parrinello-Rahman algorithm and the temperature *T* was controlled by a Nose-Hoover thermostat with a time-step of 5 femtoseconds. The system was heated up to a temperature of 2500 K and well equilibrated for 200 ps, which was supposed to be long enough to equilibrate the melts. With a cooling rate of 10^13^ K/s, the system was then cooled to obtain the metallic glasses. After being cooled down to 300 K, the system was allowed to evolve for 200 ps. When the system was fully equilibrated at the given temperature, the ultimate configurations of the (NiTi)_90_Mo_10_, (NiTi)_80_Mo_20_ and (NiTi)_70_Mo_30_ MGs were obtained.

### Uniaxial tensile test

To study the effects of the spatial network on the mechanical properties of MGs, the uniaxial tensile tests commonly used in laboratory experiments were employed in the present work. During the tensile tests, the nanopillars have a length of 30 nm and diameter of 4 nm (~25000 atoms), 6 nm (~55000 atoms), 8 nm (~100000 atoms), 10 nm (~155000 atoms), respectively. The nanopillars were cut from large initial samples, which were obtained by replication of the 4000-atom configurations from the as-quenching sample or *fcc* solid solution model. After the nanopillars were relaxed to energy-minimizing positions, uniaxial tensile loading was applied until failure. The nanopillars were loaded in tension in the *x* direction by applying a ramp velocity that went from zero in the middle to the same maximum value at the two loading ends. The purpose of the initial ramp velocity was to mitigate effects from shock loading that can occur in dynamic loading conditions^52^. The Nose-Hoover barostat and thermostat were applied to control the pressure and temperature, respectively. MD simulations proceeded at selected temperatures (100, 200, and 300 K) with a time step of 1 femtosecond.

During the simulations, the structural transitions in the models are monitored by the structure factor *S*(*q*), which can be calculated by^53^


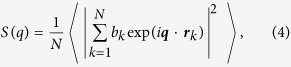


where 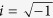, *N* is the number of atoms, and ***q*** is scattering vector. *b*_*k*_ and ***r***_*k*_ are the scattering length and position vector of atom *k*, respectively. It should be noted that the *S*(*q*) calculated by Eq. (4) is not normalized. Meanwhile, the alloy structures were further analyzed by the three-dimensional (3-D) atomic configurations, Honeycutt-Anderson pair analysis^54^, Voronoi tessellation methods^55^ and so on.

To quantify the local distortion at atomic-scale, the atomic local shear strain 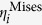 for each atom *i* are introduced^56^. Calculation of 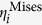 requires two atomic configurations, *i*.*e*., one current and one reference. First, a local transformation matrix ***J***_*i*_ is constructed and best maps


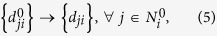


where ***d***′s are vector separations (row vectors) between atom *j* and *i* (superscript 0 means the reference configuration). Here, *j* is one of atom *i*′s nearest neighbors, and 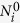 is the total number of nearest neighbors of atom *i*, at the reference configuration. ***J***_*i*_ is determined by minimizing^57^


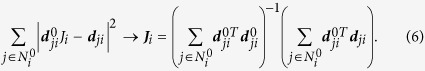


For each ***J***_*i*_, the local Lagrangian strain matrix is computed as


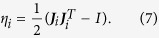


We can then define atom *i*′s local shear invariant^48^ as


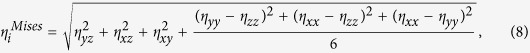


## Additional Information

**How to cite this article**: Yang, M. H. *et al*. Proposed correlation of structure network inherited from producing techniques and deformation behavior for Ni-Ti-Mo metallic glasses *via* atomistic simulations. *Sci. Rep.* **6**, 29722; doi: 10.1038/srep29722 (2016).

## Supplementary Material

Supplementary Information

## Figures and Tables

**Table 1:** Honeycutt-Andersen (H-A) analysis for the (NiTi)_100−*x*_Mo_*x*_ MGs obtained by LMQ (Liquid melt quenching) and SSA (Solid-state amorphization).

	Methods	*ico*1551	*dico*1431	*dico*1541	*bcc*1661	*bcc*1441	*fcc/hcp*142
(NiTi)_90_Mo_10_	LMQ	12.341	22.971	13.026	2.214	1.772	15.648
	SSA	11.667	22.592	12.544	1.833	1.496	15.211
(NiTi)_80_Mo_20_	LMQ	14.831	21.933	15.255	2.819	2.535	14.453
	SSA	13.428	21.133	14.149	3.034	2.550	13.851
(NiTi)_70_Mo_30_	LMQ	17.452	21.689	16.853	3.846	3.185	13.385
	SSA	15.813	21.605	15.474	3.695	3.133	13.923

The *ico* denotes icosahedral-like bond pairs with an index of 1551, *bcc* denotes *bcc*-like bond pairs with indices of 1661 or 1441, *dico* indicates distorted icosahedral-like bond pairs of 1541 or 1431, and *fcc/hcp* indicates *fcc*- or *hcp*-like bond pairs of 1421 or 1422, and here 142 is used for abbreviation.
