# Supplementary material for: Proposed correlation of structure network inherited from producing techniques and deformation behavior for Ni-Ti-Mo metallic glasses via atomistic simulations
Source: Sci Rep. 2016 Jul 15;6:29722. doi: 10.1038/srep29722 (PMC4945915; doi:10.1038/srep29722)
Supplement: Supplementary Information [file srep29722-s1.pdf]

Supplementary materials for

Proposed correlation of structure network inherited from producing techniques and deformation behavior for Ni-Ti-Mo metallic glasses *via* atomistic simulations

M.H. Yang, J.H. Li* and B.X. Liu

This PDF file includes:

1. Construction of Ni-Ti-Mo interatomic potential
2. Metallic glass formation for Ni-Ti-Mo system
3. Size-dependence of simulated stress-strain curves
4. Schematic diagram of spatial connectivity between the dominant clusters
5. Supplementary Figure S1 to S5
6. Supplementary Table S1 to S3
7. Reference

* Corresponding author: lijiahao@mail.tsinghua.edu.cn.

1. Construction of Ni-Ti-Mo interatomic potential

To develop an atomistic approach, the construction of a realistic interatomic potential of an alloy system is of critical importance. To the best of the authors' knowledge, no interatomic potential has been published for the Ni-Ti-Mo system. In the present study, a set of the Ni-Ti-Mo interatomic potentials are constructed under a formulism proposed recently by the authors' group, *i.e.*, the new long-range empirical potential (LREP) scheme [1].

The proposed potential could distinguish the energy differences between stable and hypothetic structures of *bcc*, *fcc* and *hcp* metals. In particular, it can distinguish the energy difference between the *fcc* and *hcp* structures. Moreover, the total energy and force derived from the proposed potential could keep continuous and smooth in the entire calculation range. Thus, it can properly resolve the cutoff problem without a truncation function [2]. According to the potential, the potential energy E_i of atom i can be calculated as follows:

$$E_i = \frac{1}{2}\sum_{j\neq i} V(r_{ij}) - \sqrt{\sum_{j\neq i}\phi(r_{ij})}, \tag{S1}$$

where the whole second term of *Eq.* S1 is the cohesive part and r_{ij} is the distance between atoms i and j of the system at equilibrium. $V(r_{ij})$ is the pair part and $\phi(r_{ij})$ is the electron density part. These items can be expressed as follows:

$$V(r_{ij}) = (1 - r_{ij}/r_{c1})^m (c_0 + c_1 r_{ij} + c_2 r_{ij}^2 + c_3 r_{ij}^3 + c_4 r_{ij}^4), 0 < r_{ij} \le r_{c1}, \tag{S2}$$

$$\phi(r_{ij}) = \alpha^2 (1 - r_{ij}/r_{c2})^n, 0 < r_{ij} \le r_{c2}, \tag{S3}$$

where r_{c1} and r_{c2} are the cutoff radii for pair part and electron density part, respectively. The exponent m and n are two integers and adjustable according to the

specific metals; α and c_i are the potential parameters to be determined by a fitting procedure.

Concerning the interactions between atoms, there should be six sets of potential parameters in the Ni-Ti-Mo ternary system, *i.e.*, three sets for the interactions of pure metal Ni-Ni, Ti-Ti and Mo-Mo, and three sets for the cross interactions of Ni-Ti, Ni-Mo and Ti-Mo. Generally, the potential [3] for Ni-Ni, Ti-Ti and Ni-Ti as well as the potential [4] for Ni-Ni, Mo-Mo and Ni-Mo were originally developed by Li *et al*. Therefore, the cross interaction for Ti-Mo should be determined by fitting to the basic physical properties of the intermetallic compounds with various structures and compositions. In order to acquire enough physical properties of the related compounds, the first-principle calculations were performed by using the Cambridge serial total energy package (CASTEP) in Materials Studio[5,6]. In the present work, the exchange and correlation items were described by the generalized-gradient approximation (GGA) of Perdew and Wang (PW91)[7], and the ion-electron interactions were treated by the projector augmented wave (PAW) method [8]. The cutoff energy was chosen to be 700.0 eV, and the Brillouin-zone was sampled using the Monkhorst-Pack method[9] with nearly constant *k*-point densities for each calculation.

Now we present the detailed fitting results of the Ni-Ti-Mo interatomic potential. The six sets of potential parameters are summarized in Table S1. As shown in Table S2, it lists the lattice constants, bulk modulus and elastic modulus of B2 and $D0_3$ Ti-Mo compounds derived from the potential and the first-principle calculation

developed by Ikehata *et al* [10]. It can be seen that the potential derived lattice constants, bulk modulus and elastic modulus of Ti-Mo compounds match well with the previous study. Table S3 shows the lattice constants, formation energies, and bulk moduli of related intermetallic compounds in the Ti-Mo binary system derived from potential. Comparing with the first-principle calculation, the maximum error of the cohesive energies and lattice constants is less than 4%, confirming that the constructed potential could well derive the structure and energy of these compounds in the systems.

To further evaluate the validity of the constructed potential, another approach is to check whether the potential can describe atomic interactions under non-equilibrium states. Therefore, we obtain the equation of state (EOS) from the constructed potential and then compared it with the Rose equation, which has been proved to be universal for most categories of solids[11]. Fig. S1(a) and Fig. S1(b) plot the pair parts, the cohesive parts and the potential energies as a function of the lattice constants calculated from the constructed potential, and the corresponding Rose equations for $L1_2$ $TiMo_3$, $L1_2$ Ti_3Mo, $D0_{19}$ $TiMo_3$ and $D0_{19}$ Ti_3Mo; B2 TiMo, $D0_3$ $TiMo_3$ and $D0_3$ Ti_3Mo. It can be seen that the pair parts, cohesive parts and the potential energy of these intermetallic compounds keep smooth and continuous in the entire range, and the EOS derived from the proposed potential agree well with the corresponding Rose equation. Meanwhile, there are not any discontinuities in energy as well as the force calculated from the constructed potential for the related compounds, which can avoid unphysical behavior in Molecular Dynamics and Monte Carlo simulations. It follows

that the newly constructed long-range empirical potential can reasonably describe the atomic interactions of the Ni-Ti-Mo system even far from the equilibrium state, thus providing additional evidence that the constructed potentials can be related to the structure and energy of the system.

As the stable crystalline structures of Ni, Ti and Mo are *fcc*, *hcp* and *bcc*, respectively, three types of solid solution models, *i.e.*, the *fcc*, *hcp* and *bcc* solid solution models, were constructed based on the main component of the alloy composition. For both *fcc* and *bcc* models, the [100], [010] and [001] crystalline directions are parallel to the x, y and z axes, respectively, whereas for the hcp model, the [100], [120] and [001] crystalline directions are parallel to the x, y and z axes. Meanwhile, we varied x and y with a composition interval of 5% to construct the $Ni_xTi_yMo_{1-x-y}$ solid solution models over the entire composition triangle of the system.

2. Metallic glass formation for Ni-Ti-Mo system

2.1. Glass formation region of Ni-Ti-Mo system

Considering the total structure factor $S(q)$ and atomic position projections of each specific alloy, the Ni-Ti-Mo composition triangle is divided into five regions by six critical solid solubility lines, and the metallic glass forming composition diagram is constructed in Fig. S2. When an alloy composition is situated beyond the line KJ and moving towards the Ni corner, or beyond line GHI and moving towards the Ti-Mo side, the solid solution structures can remain stable, and these two regions are

consequently classified as the crystalline regions. When the composition of the alloy moves from the lines ABCD and EF towards the central hexagonal region enclosed by ABCDEF, the solid solution structure becomes unstable and collapses, giving rise to the formation of the amorphous phase. This region is thus defined as the amorphous region, *i.e.*, the GFR of the Ni-Ti-Mo system. Between the crystalline regions and the amorphous region, there exist ordered-disordered transitional regions, within which the amorphization occurs partly, thereby transforming into a state featuring the ordered-disordered coexisting structure. Considering the completely miscible Ti-Mo equilibrium phase diagram, it would be difficult to obtain amorphous alloys along the Ti-Mo side. This is in accordance with the crystalline region in the Ti-Mo side and the amorphous region in the central hexagonal region, as is exhibited in Fig. S2.To validate the amorphous region, it is of great importance to compare the predicted GFR of the Ni-Ti-Mo system with the experimental observations. Various experimental results were collected and marked by different symbols, which mostly fall within the hexagonal region. It can be seen that ternary $(NiTi)_{80}Mo_{20}$ MGs on the HG line could be synthesized by IBM, of which the composition marked by red triangle is located within the shaded area (GFR). From the discussion above, all the experimental observations suggest that the predictions by MD simulations for the Ni-Ti-Mo system are quite reasonable.

2.2. Optimization of glass formation compositions

According to the MD simulation results, one can conveniently predict the possibility of metallic glass formation in the Ni-Ti-Mo system at a given composition.

Nevertheless, there are still issues related to evaluating the GFA of Ni-Ti-Mo alloys at different compositions, and pinpoint the optimal alloy composition sub-region with the highest GFA in the glass formation region. From a thermodynamics viewpoint, the formation enthalpy difference between the amorphous phase and the solid solution could serve as the driving force for amorphization. One can evaluate the GFA of the Ni-Ti-Mo system by calculating the driving force for amorphization at a given alloy composition, *i.e.*, the larger the driving force, the higher the GFA and the easier the amorphous alloys can be formed.

Assume that E_{am} is the energy per atom of the $Ni_xTi_yMo_{1-x-y}$ amorphous phase and E_{Ni}, E_{Ti}, and E_{Mo} are the lattice energies[12,13]of Ni, Ti, and Mo atoms in ground state, respectively. The formation enthalpy for the amorphous phase ΔE^{am}, which have been calculated in the MD simulations, can be expressed by

$$\Delta E^{am} = E_{am} - [xE_{Ni} + yE_{Ti} + (1-x-y)E_{Mo}]. \tag{S4}$$

Meanwhile, an efficient and relevant MC simulations were performed to compute the formation energy of the solid solutions, *i.e.*, $\Delta E^{s.s}$. Assume that $E_{s.s}$ is the energy per atom of the $Ni_xTi_yMo_{1-x-y}$ solid solutions, and the formation enthalpy $\Delta E^{s.s}$ of the $Ni_xTi_yMo_{1-x-y}$ solid solutions can then be expressed by

$$\Delta E^{s.s} = E_{s.s} - [xE_{Ni} + yE_{Ti} + (1-x-y)E_{Mo}]. \tag{S5}$$

Therefore, the formation enthalpy difference between the amorphous phase and the solid solution can be expressed by

$$\Delta E^{am\text{-}s.s} = E_{am} - E_{s.s}, \tag{S6}$$

where the $\Delta E^{am-s.s}$ is defined as the driving force for amorphization .

Based on the results from the MD and MC simulations, the contour map of the amorphization driving force for Ni-Ti-Mo system is plotted in Fig. S3(a), as well as the driving force for the $(NiTi)_{100-x}Mo_x$ alloys along the HG line is shown in Fig. S3(b). From the Fig. S3(a), the $\Delta E^{am\text{-}s.s}$ is negative over the whole GFR, indicating that the energy of the amorphous phase is lower than that of the solid solution, thus formation of the amorphous phase is energetically favored. Besides, the larger the energy difference, the stronger the driving force for glass formation. Further inspecting Fig. S3(a), it can be found that the alloy composition represented by red dots features a lower $\Delta E^{am\text{-}s.s}$ than any other composition regions, indicating that their driving forces for amorphization are stronger. We define this composition sub-region as the optimal compositions for Ni-Ti-Mo metallic glass formation, within which the alloys have greater GFA than those alloys located outside the sub-region. Therefore, it provides basic guidelines to design appropriate alloy compositions for producing Ni-Ti-Mo metallic glasses. As shown in Fig. S3(b), it is found that the driving force of the $(NiTi)_{100-x}Mo_x$ alloys first increases gradually by adding the appropriate Mo concentration, and then after reaching the peak value, decreases with further increasing of Mo. Once the addition of Mo is higher than 66 at.%, the solid solution could maintain its crystalline lattice and no unique amorphous phases would be formed, indicating that the GFR of $(NiTi)_{100-x}Mo_x$ is 0-66 at.%.

3. Size-dependence of simulated stress-strain curves

As shown in Fig. S4, the stress-strain curves of the nanopillars with a length of 30 nm and diameter of 4, 6, 8, 10 nm are displayed, respectively. For the nanopillar of 8 nm and 10 nm diameter, the σ_{over} value of $(NiTi)_{80}Mo_{20}$ MGs obtained by LMQ is larger than that obtained by SSR. When the diameter becomes 6 nm, the stress-strain curves obtained by both producing methods are almost the same. However, the sample obtained by SSR becomes more brittle after the diameter decreases to 4 nm. Therefore, the reduction diameter of the nanopillar can result in material brittleness. However, the understanding of size-dependent/independent deformation behavior of BMGs at low temperature still needs further researches. It has been reported that the decreasing specimen size can even induced tensile ductility and enhancing yield strength [14-16], which can be attributed to the smaller sample size than the shear-band spacing and the equivalent critical shear offset [17]. On the contrary, some other literature [18-21] reported size-independent deformation behavior for the BMGs at room temperature, namely the yield strengths and the plastic deformation modes are insensitive to the specimen sizes and initial structural states.

4. Schematic diagram of spatial connectivity between the dominant clusters

The spatial structure networks are made of dominant clusters that interconnect to neighbor dominant clusters by sharing their vertex, edge, face or volume. As shown in Fig. S5(a), there are six dominant clusters highlighted with dashed circles in the

supercluster, and the dominant clusters are connected with the neighboring clusters by vertex, edge, face and volume linkages. This spatial structure network stretches in space and could serve as the backbone of the MG structure over an extended range. As an example, Fig. S5(b) shows the spatial distributions of a typical cross-linked patch containing 47 dominant clusters extracted from $(NiTi)_{80}Mo_{20}$ MGs. By assessing the structures of the model alloy, only the centered atoms of the dominant clusters are displayed and marked by different colors, indicating how the dominant clusters are linked with different numbers of neighboring clusters to form the spatial structure network.

5. Supplementary Figure

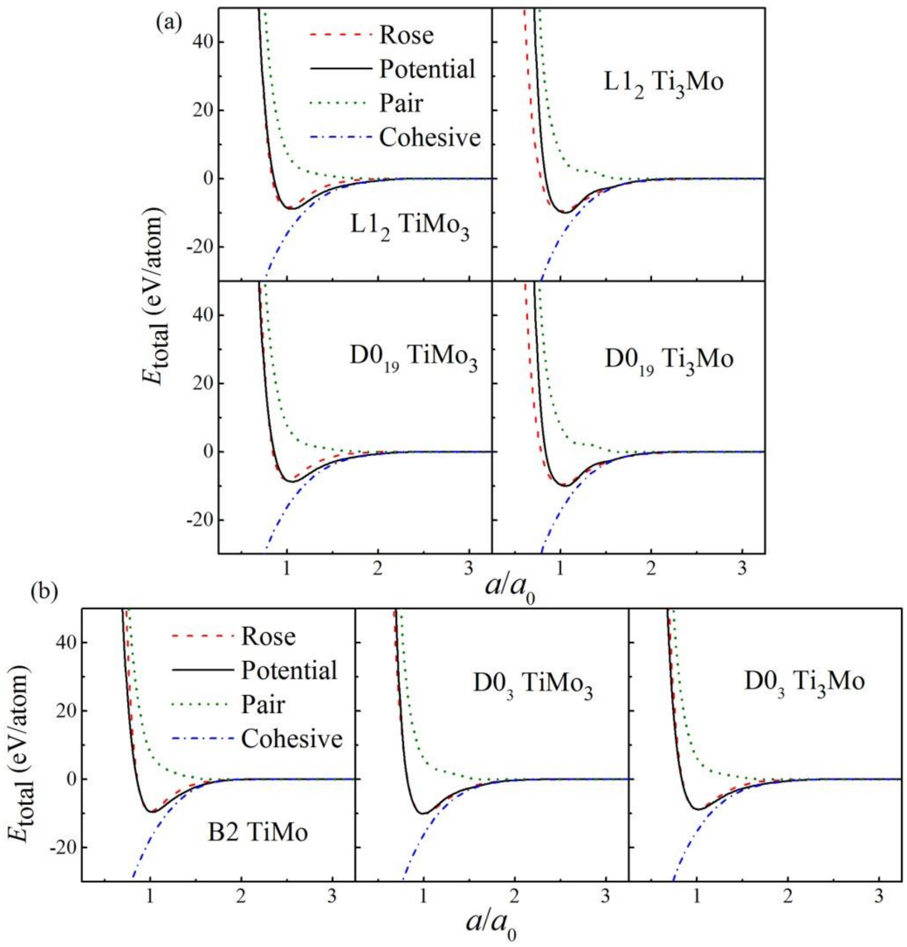

Supplementary Figure S1. The pair, cohesive and potential energies as a function of the lattice constants and the corresponding Rose equation for (a): L1_2 $TiMo_3$, L1_2 Ti_3Mo, D0_{19} $TiMo_3$ and D0_{19} Ti_3Mo; (b): B2 TiMo, D0_3 $TiMo_3$ and D0_3 Ti_3Mo.

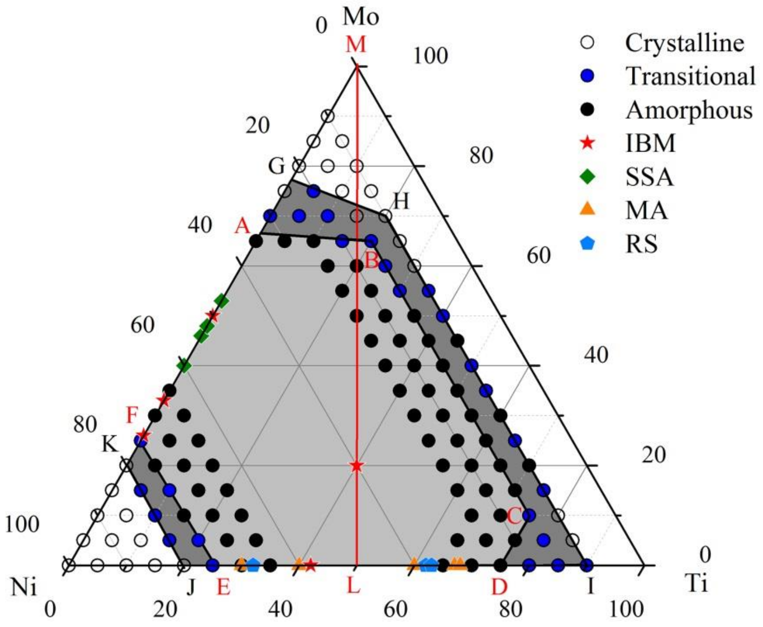

Supplementary Figure S2. The glass formation region (gray shaded area) derived from MD simulations at 300 K with different experimental data for the Ni-Ti-Mo system. Solid-state amorphization is abbreviated as SSA, ion beam mixing as IBM, rapid solidification as RS, and mechanical alloying as MA.

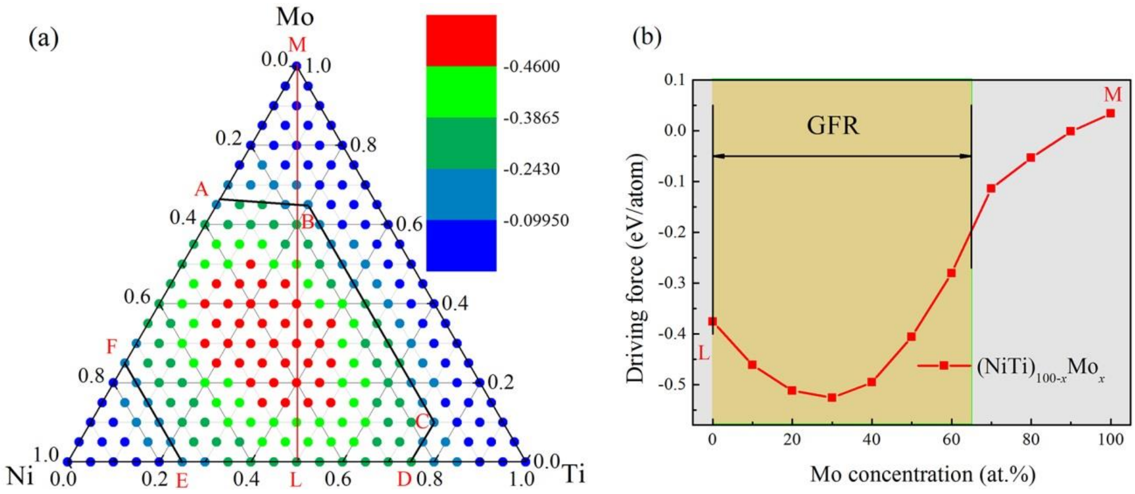

Supplementary Figure S3. The driving force for the crystalline-to-amorphous transition of the Ni-Ti-Mo system calculated from MC simulations (a), and the driving force of the $(NiTi)_{100-x}Mo_x$ alloys for amorphization (b).

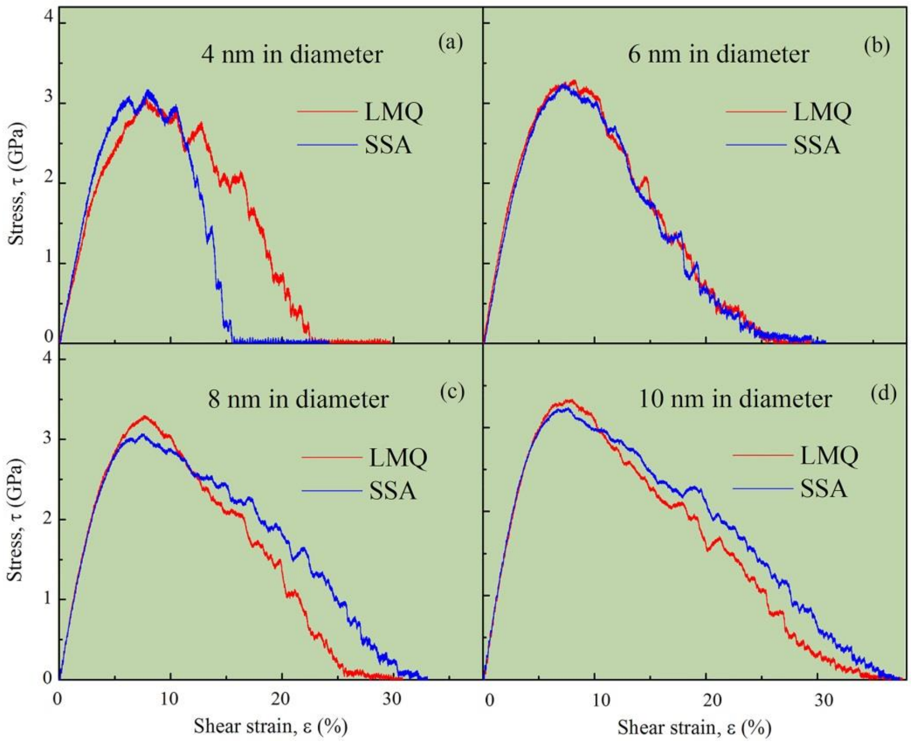

Supplementary Figure S4. The strain-stress curves for nanopillars with a length of 30 nm and different diameters at 100 K: (a) 4 nm, containing ~25000 atoms; (b) 6 nm, containing ~55000 atoms; (c) 8 nm, containing ~100000 atoms; (d) 10 nm, containing ~155000 atoms.

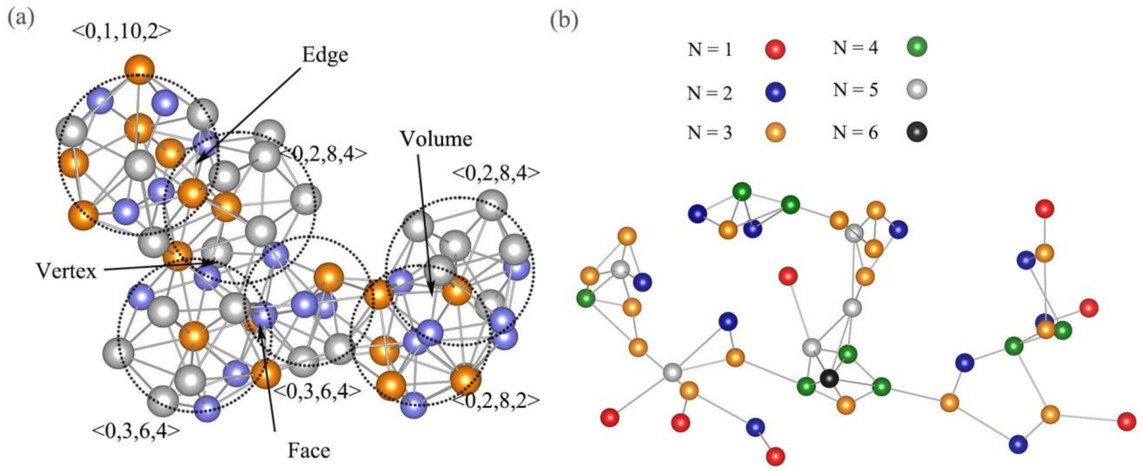

Supplementary Figure S5. A supercluster consisting of 6 dominant clusters, *i.e.*, <0, 2, 8, 4>, <0, 3, 6, 4>, <0, 2, 8, 2>, <0, 1, 10, 2> and the icosahedron <0, 0, 12, 0>, highlighted with dashed circles. The Ni, Ti, and Mo atoms are colored blue, gray and orange, respectively (a). The connection mode of the dominant cluster network in the extended scale is revealed by a cross-linked patch containing 47 clusters with different *N*. Only the center atoms of the dominant cluster are plotted as points (b).

6. Supplementary Table

Supplementary Table S1. The parameter of the constructed potential for Ni-Ti-Mo system.

	Ni-Ni [3,4]	Ti-Ti [3]	Mo-Mo [4]	Ni-Ti [3]	Ni-Mo [4]	Ti-Mo
m	4	4	4	4	4	4
n	5	4	6	5	6	5
r_{c1} (Å)	5.7689	5.7959	4.7577	4.4203	5.2447	4.9559
r_{c2} (Å)	7.2452	6.9572	7.2424	7.8189	5.0170	5.4064
c_0 (eV)	313.3092	387.5553	10271.2947	584.7520	2297.9742	1621.6550
c_1 (eV/Å)	-381.1370	-439.8156	-14753.9697	-633.0180	-3065.9503	-2015.4381
c_2 (eV/Å^2)	172.5236	192.9678	7943.7440	276.7508	1548.7581	1007.2635
c_3 (eV/Å^3)	-34.5074	-386.9957	-1896.3805	-62.3333	-351.4804	-233.7983
c_4 (eV/Å^4)	2.5963	3.0186	169.3338	7.0591	30.2669	21.0000
α (eV)	4.0913	5.4307	-11.8289	8.2235	-19.1951	-22.5323

Supplementary Table S2. Physical properties (lattice constants (Å), elastic constants (Mbar) and bulk modulus (Mbar)) of B2 and $D0_3$ compounds obtained from constructed potential (first line) and the first-principles calculations developed by Ikehata *et al.* [10] (second line).

	Structure	a	C_{11}	C_{12}	C_{44}	B_0
$Ti_{0.75}Mo_{0.25}$	Fit. $D0_3$	3.333	1.607	1.566	0.878	1.579
	Cal. $D0_3$	3.273	1.605	1.256	0.341	1.372
$Ti_{0.5}Mo_{0.5}$	Fit. B2	3.393	2.091	1.756	0.970	1.868
	Cal. B2	3.280	2.240	1.466	0.104	1.724
$Ti_{0.25}Mo_{0.75}$	Fit. $D0_3$	3.300	2.592	1.674	0.890	1.980
	Cal. $D0_3$	3.306	3.636	1.515	0.620	2.222

Supplementary Table S3. Physical properties (lattice constants (Å), cohesion energies (eV) and bulk moduli (MBar)) of $L1_2$ and $D0_{19}$ compounds obtained from constructed potential (first line) and the first-principles calculations (second line).

	Ti_3Mo	$TiMo_3$	Ti_3Mo	$TiMo_3$
Structure	$L1_2$	$L1_2$	$D0_{19}$	$D0_{19}$
a or *a*, *c*	3.975	4.094	5.607, 4.613	5.670, 5.020
	4.054	4.014	5.738, 4.690	5.466, 5.005
E_c	5.780	5.456	5.776	5.441
	5.735	5.490	5.737	5.550
B_0	1.432	1.930	1.538	1.854
	1.399	2.038	1.511	1.928

References:

1. Dai, X., Kong, Y. & Li, J. Long-Range Empirical Potential Model: Application to Fcc Transition Metals and Alloys. *Phys. Rev. B*. **75**, 104101 (2007).

2. Frenkel, D. & Smit, B. *Understanding Molecular Simulation:From Algorithms to Applications*(Academic press, New York) 2001.

3. Li, Y., Li, J., Liu, J. & Liu, B. Atomic Approach to the Optimized Compositions of Ni–Nb–Ti Glassy Alloys with Large Glass-Forming Ability. *RSC Adv*. **5**, 3054-3062 (2015).

4. Li, Y., Luo, S. Y., Li, J. H., Liu, J. B. & Liu, B. X. Interatomic Potential to Predict the Glass-Forming Ability of Ni–Nb–Mo Ternary Alloys. *J Mater Sci*. **49**, 7263-7272 (2014).

5. Clark, S. J. et al. First Principles Methods Using Castep. *Z. Kristallogr. - Cryst. Mater.* **220**, 567-570 (2005).

6. Segall, M. D. et al. First-Principles Simulation: Ideas, Illustrations and the Castep Code. *J. Phys.: Condens. Matter.* **14**, 2717 (2002).

7. Perdew, J. P. & Wang, Y. Function and its Coupling-Constant Average for the Spin-Polarized Electron Gas John P. *Phys. Rev. B*. **46**, 12947-12954 (1992).

8. Blochl, P. E. Projector Augmented-Wave Method. *Phys. Rev. B*. **50**, 17953-17979 (1994).

9. Monkhorst, H. J. & Pack, J. D. Special Points for Brillouin-Zone Integrations. *Phys. Rev. B*. **13**, 5188-5192 (1976).

10. Ikehata, H. et al. First-Principles Calculations for Development of Low Elastic Modulus Ti Alloys. *Phys. Rev. B*. **70**, 174113 (2004).

11. Rose, J. H., Smith, J. R., Guinea, F. & Ferrante, J. Universal Features of the Equation of State of Metals. *Phys. Rev. B*. **29**, 2963-2969 (1984).

12. Lide, D. R. & Bruno, T. J. *Crc Handbook of Chemistry and Physics*(CRC press, New York) 2012.

13. Kittel, C. & P, M. *Introduction to Solid State Physics*(Wiley, New York) 1996.

14. Lee, C. J., Huang, J. C. & Nieh, T. G. Sample Size Effect and Microcompression of $Mg_{65}Cu_{25}Gd_{10}$ Metallic Glass. *Appl. Phys. Lett.* **91**, 161913 (2007).

15. Wu, W. F., Han, Z. & Li, Y. Size-Dependent “Malleable-to-Brittle” Transition in a Bulk Metallic Glass. *Appl. Phys. Lett.* **93**, 61908 (2008).

16. Lai, Y. H. et al. Bulk and Microscale Compressive Behavior of a Zr-Based Metallic Glass. *Scripta Mater*. **58**, 890-893 (2008).

17. Wu, F. F., Zhang, Z. F. & Mao, S. X. Size-Dependent Shear Fracture and Global Tensile Plasticity of Metallic Glasses. *Acta Mater*. **57**, 257-266 (2009).

18. Liu, M. C. et al. Is the Compression of Tapered Micro- And Nanopillar Samples a Legitimate Technique for the Identification of Deformation Mode Change in Metallic Glasses? *Scripta Mater*. **66**, 817-820 (2012).

19. Dubach, A., Raghavan, R., Lo Ffler, J., Michler, J. & Ramamurty, U. Micropillar Compression Studies On a Bulk Metallic Glass in Different Structural States. *Scripta Mater*. **60**, 567-570 (2009).

20. Wu, X. L., Guo, Y. Z., Wei, Q. & Wang, W. H. Prevalence of Shear Banding in Compression of Zr41Ti14Cu12.5Ni10be22.5 Pillars as Small as 150Nm in Diameter. *Acta Mater*. **57**, 3562-3571 (2009).

21. Schuster, B. E., Wei, Q., Hufnagel, T. C. & Ramesh, K. T. Size-Independent Strength and Deformation Mode in Compression of a Pd-Based Metallic Glass. *Acta Mater*. **56**, 5091-5100 (2008).
